# Task shifting of triage to peer expert informal care providers at a tertiary referral HIV clinic in Malawi: a cross-sectional operational evaluation

**DOI:** 10.1186/s12913-017-2291-3

**Published:** 2017-05-09

**Authors:** Megan Landes, Courtney Thompson, Edson Mwinjiwa, Edith Thaulo, Chrissie Gondwe, Harriet Akello, Adrienne K. Chan

**Affiliations:** 1 Dignitas International-Malawi Country Program, Zomba, Malawi; 2 Department of Emergency Medicine, University Health Network, Toronto, Canada; 3 Department of Family and Community Medicine, University of Toronto, Toronto, Canada; 4 Division of Infectious Diseases, Department of Medicine, University of Toronto, Toronto, Canada; 5 London School of Tropical Medicine and Hygiene, London, UK; 6 Tisungane Clinic, Zomba Central Hospital, Malawi Ministry of Health, Zomba, Malawi; 7 Division of Infectious Diseases, Sunnybrook Health Sciences Centre, Toronto, Canada; 8 Institute for Health Policy, Management and Evaluation and Division of Clinical Public Health, Dalla Lana School of Public Health, University of Toronto, Toronto, Canada

**Keywords:** Task-shifting, Expert patients, Lay health workers, HIV, Malawi, Triage

## Abstract

**Background:**

HIV treatment models in Africa are labour intensive and require a high number of skilled staff. In this context, task-shifting is considered a feasible alternative for ART service delivery. In 2006, a lay health cadre of expert patients (EPs) at a tertiary referral HIV clinic in Zomba, Malawi was capacitated. There are few evaluations of EP program efficacy in this setting. Triage is the process of prioritizing patients in terms of the severity of their condition and ensures that no harmful delays occur to treatment and care. This study evaluates the safety of task-shifting triage, in an ambulatory low resource setting, to EPs.

**Methods:**

As a quality improvement exercise in April 2010, formal triage training was conducted by adapting the World Health Organization Emergency Triage Assessment and Treatment Triage Module Guidelines. A cross sectional observation study was conducted 2 years after the intervention. Triage assessments performed by EPs were repeated by a clinical officer (gold standard) to assess sensitivities, specificities, positive and negative predictive values for EP triage scores. Proportions were calculated for categories of disposition by stratifying by EP and clinician triage scores.

**Results:**

A total of 467 patients were triaged by 7 EPs and re-triaged by clinical officers. With combined triage scores for *emergency* and *priority* patients we report a sensitivity of 85% and specificity of 74% for the EP scoring, with a low positive predictive value (41%) and a high negative predictive value (96%). We calculate a serious miss rate of EP scoring (i.e. missed *priority* or *emergency* patients) as 2.2%. Admission rates to hospital were highest among those patients triaged as *emergency* cases either by the EP’s (21%) or the clinicians (83%). Fewer patients triaged as *priority* by either EPs (5%) or clinicians (15%) were admitted to hospital, however these patients had the highest prevalence of same day lab testing and/or specialty referral.

**Conclusions:**

Our study provides reassurance that in the context of adequate training and ongoing supervision, task-shifting triage to lay health care workers does not necessarily lead to less accurate triaging. EPs have a tendency to be more conservative in over-triaging patients.

**Electronic supplementary material:**

The online version of this article (doi:10.1186/s12913-017-2291-3) contains supplementary material, which is available to authorized users.

## Background

In the wake of an intensifying Human Immunodeficiency Virus (HIV) pandemic over the past 20 years, low-income countries (LICs) with high HIV prevalence and concomitant frontline health worker shortages have faced critical human resources for health (HRH) crises. Some LICs have piloted and scaled approaches to address this growing crisis by involving the transfer of expert knowledge on HIV-testing, treatment initiation and treatment support from nurses and clinicians to non-clinical, lay, and peer caregivers [1, 2]. An example of this is in the development of the “expert patient” (EP), a term used to refer to people living with long-term health conditions who are provided with additional training to perform various healthcare tasks such as administration or patient counseling and education [3]. In part an endorsement of the 1994 Declaration of Greater Involvement of People Living with HIV/AIDS (PLHIV) [3], as well as a reflection of increasing trends towards patient self-management in high-resource settings [4], EP programs are being implemented as an efficient way to ensure that the ‘care continuum’ for PLHIV is being addressed.

Expert patients that are PLHIV have been increasingly involved in filling various roles within antiretroviral therapy (ART) clinics and services, such as HIV counseling and testing [5], assisting with ART registration, tracing patients lost to follow-up [6], facilitating support groups, adherence counseling and support, and collecting and collating statistics [7]. The current literature encompasses various aspects of assessing these programs including efficiency, cost-savings, and safety of a range of tasks [8–10], However, the meaning and utility of patient ‘expertise’ in this setting differs markedly from the biosocial underpinnings of the traditional EP model that envisioned the creation of instrumental therapeutic ties based on shared lived experience and successful self-management of a specific condition [3]. Unlike their counterparts in middle- and high income countries, EPs that are PLHIV and acting within a context of extreme economic scarcity, are often valued the most by the health system for their task-shifted roles in mitigating chronic and critical HRH shortages.

While there is descriptive literature [11, 12], capturing the spectrum of EP programs adopted by various non-governmental organization (NGO) and public sector programs in high HIV prevalence, low resource settings, as well as qualitative studies capturing the EP experience from a patient, health care worker and EP perspective [13–15], there are very few evaluations of EP program efficacy in this setting [16–20].

One task that is often delegated informally to EPs working in facility-based settings is triage. Triage is the process of sorting patients in terms of their priority based on the severity of their condition and ensures that no harmful delays occur to potential lifesaving treatments. Most ART clinics in high HIV prevalence, low resource settings work on a first come, first serve basis, however they often see patients with acute illness, making a functioning triage system a critical component of quality in service delivery. We sought to evaluate the capacitation of EPs to conduct task-shifted patient triage as a quality improvement initiative of an existing EP program at an ambulatory and tertiary referral HIV clinic in Malawi.

## Methods

### Study setting and description of the intervention

Zomba District (population 670,533) is situated in the southeastern region of Malawi [21], and has one of the highest estimated adult HIV prevalence rates in the country (14.5%) [22]. Roll out of ART in Zomba District began in 2004 by the Malawi Ministry of Health (MOH) and with the support of Dignitas International, a Canadian NGO, “Tisungane Clinic” at Zomba Central Hospital was established as a tertiary referral HIV [7]. At the time of this study, over 14,500 patients had been enrolled on ART, and HIV services had been decentralized to 24 primary health centers to facilitate patient access to treatment and also decongest the referral clinic [23].

During the period of evaluation, Tisungane Clinic assessed 100–150 HIV+ adults and children daily. Patients registered for both the pre-ART and ART programs attended clinic for initiation of treatment, routine follow-up and with acute intercurrent illness. The EP program at Tisungane Clinic was started in 2007 and has been previously described by Tenthani et al. [24] EPs at the time of study, had been already trained in the Malawi Ministry of Health standardized curricula on ART initiation and refresher counselling, as well as adherence counselling. EPs had also been trained and capacitated through supportive supervision and on-the-job mentorship by clinicians, nurses and registration clerks to manage patient flow in the clinic and were task-shifted light clinical tasks including taking and recording vital signs and anthropometry. The majority of patients arrived for clinic were organized by EPs in the order of their arrival to see the nurses for drug dispensation and initial assessments and then referred onwards to clinicians for complicated visits and ART initiations. Due to the patient volume, wait times could often be for several hours, even to see the nurse for an initial symptom assessment and triage. As this was an outpatient ambulatory clinic, wait times are not formally assessed. An informal triage system had developed over time by the EPs to flag patients presenting with acute illness and bypass others who were less ill, to be seen by the clinicians directly. This informal system helped to facilitate early admission and also the opportunity for rapid referral for laboratory testing, radiography and specialist assessment.

As a quality improvement exercise in April 2010, formal triage training was conducted by adapting the World Health Organization (WHO) Emergency Triage Assessment and Treatment (ETAT) Triage Module Guidelines [25], and translating tools into English and Chichewa (the local language; see Additional file 1). Seven expert patients completed triage training consisting of a full-day workshop, a review session and on-the- job mentorship by the 2 physician trainers over two weeks. EPs were trained on how to categorize both adult and pediatric patients as *emergency* (i.e. life-threatening signs or symptoms needing immediate assessment by a clinician) and *priority* (i.e. non-life-threatening but still requiring urgent prioritization) cases. As patients arrived in clinic and began to queue, EPs were instructed to assess each patient and give a red card to all *emergency* cases and take them immediately to be assessed by a clinician, or give a yellow card to all *priority* patients and place them in the front of the line to see the next available clinician. All *routine* patients (everyone other than emergency and priority patients) were given a green card and placed in the nursing line in order of arrival for assessment. A separate orientation session was held with the nursing staff and the clinician officers in order to explain the triage criteria and implementation so that they could provide ongoing supervision.

### Study design

A cross-sectional operational evaluation of the EP triage system was conducted during a two-week period in March 2012. All patients presenting for either a scheduled clinician visit or an unscheduled visit for an acute intercurrent health problem were included. As per standard clinic procedures, all patients were triaged first by the expert patients who completed a standardized data collection form and then prioritized for clinician assessment based on EP triage scores. On assessment, clinicians repeated the triage process filling in a similar standardized data collection form and were additionally asked to record disposition data (i.e. discharged home, sent for same-day laboratory testing or specialist referral, or admitted to hospital). Data on demographic characteristics of expert patients was collected by standard interview scripts.

### Data management and analysis

All data were entered into an electronic database, from which standard descriptive statistics were calculated. Sensitivities, specificities, positive and negative predictive values were calculated for EP triage scores using clinician scores as the gold standard. Proportions were calculated for categories of disposition by stratifying by EP and clinician triage scores.

## Results

### Characteristics of expert patients

Seven EPs (5 female, 2 male) were included with a mean age of 35 years (min. 27, max. 45). All had been on ART for 5 or more years (maximum 9 years) and had been working at the clinic between 4 and 5.5 years. All EP’s had completed primary school, five had completed their Junior Certificate Examination (a national standardized test conducted after two years of secondary education) and all could read and write in Chichewa, but only two in English. All seven EP’s had completed the triage training and participated in triaging patients in the clinic during the data collection.

### Patient triage outcomes

A total of 467 patients presented for a clinic visit for either a scheduled or an unscheduled new health problem during the study period. Data on clinician triage scores were missing on 14 patients first triaged by the expert patients and were thus excluded from the analysis. None of these excluded patients were triaged as *emergency* by the EPs and only 5 were triaged as *priority*.

Table 1 shows the triage scores from both EPs and the clinicians (n = 453), and Table 2 describes the sensitivity, specificity, positive and negative predictive values for the EPs’ scores. Fourteen patients were triaged as *emergency* cases by the EPs; 2 were confirmed by the clinicians as *emergency* and 5 as *priority*, giving an 85% sensitivity for EPs in identifying potentially acutely ill patients i.e. combined *emergency* or *priority* patients.

**Table 1:** Expert patient and clinician scores per patient (*N* = 453)

	Clinician score			
Expert patient score	Emergency	Priority	Routine	Total
Emergency	2	5	7	14
Priority	3	59	91	153
Routine	0	8	266	274
No data available	1	3	8	12
Total	6	75	372	453

**Table 2:** Comparison of triage scores of expert patients and clinicians

	Triage score by expert patient	Triage score by clinician	Sensitivity (%)	Specificity (%)	Positive predictive value (%)	Negative predictive value (%)
Emergency	14	6	33.3	97.3	14.3	99.1
Priority	153	75	78.7	75.5	38.6	94.8
Emergency and priority combined	167	81	85.2	73.7	41.3	95.8

Additionally, for EP *emergency* scores we report a low positive predictive value (14.3%) but a high negative predictive value (99.1%) indicating the EP’s tendency to err on the side of caution and “over-triage” patients. **Four** false negative scores by the EPs were identified among *emergency* cases (i.e. not triaged as *emergency* by the EPs, but confirmed as *emergency* by the clinicians), three of whom had been triaged as *priority* by the EP and one for whom data on EP triage score was missing.

One-hundred fifty three patients were triaged as *priority* by the EPs, of which 59 were confirmed *priority* by the clinicians and giving a 78.7% sensitivity for EP scoring of *priority* cases. There were 91 (59.4%) false positive *priority* scores i.e. triaged as *priority* by the EPs, but triaged *as routine* by the clinicians.

Among the 274 patients triaged as *routine* by the EPs, 12 false negative scores were identified (i.e. not triaged as *priority* by EPs but then identified as such by clinicians); 5 were triaged at a higher level as *emergency* by EPs and 8 were under-triaged as *routine*. For EP *priority* scores, we report a similar trend as with the triaging of *emergency* patients of a low positive predictive value (PPV; 38.6%) and a high negative predictive value (NPV; 94.8%), indicating a trend towards over-triaging patients by the EPs.

Overall when looking at the EP’s combined triage scores for *emergency* and *priority* patients (i.e. all patients requiring prioritization at the clinic) we report a sensitivity of 85.2% and specificity of 73.7% for the EP scoring, with a low PPV (41.3%) and a high NPV (95.8%). We calculate a serious miss rate of EP scoring (i.e. missed *priority* or *emergency* patients) as 2.4%, as we identified 8 *routine* scores by EPs that should have been *priority* and 3 *priority* scores by EPs that should have been *emergency* (11/453).

### Disposition outcomes

Disposition data stratified by EP and clinician triage score is presented in Table 3.

**Table 3:** Disposition of patients by expert patient or clinician triage scores

Triage score	Disposition	Expert patient	Clinicians
		*N* = 441^a^	*N* = 453
		N (%)	N (%)
Emergency	Admitted to hospital	3 (21.4)	5 (83.3)
	Same-day laboratory testing	1 (7.1)	1 (16.7)
	Same-day specialist referral	0 (0)	0 (0)
	Discharged	6 (42.9)	0 (0)
	No data on disposition	4 (28.6)	0 (0)
Priority	Admitted to hospital	7 (4.6)	11 (14.9)
	Same-day laboratory testing	23 (15.0)	21 (28.4)
	Same-day specialist referral	6 (3.9)	2 (2.9)
	Discharged	107 (70.0)	30 (42.9)
	No data on disposition	10 (6.5)	10 (14.3)
Routine	Admitted to hospital	1 (0.4)	0
	Same-day laboratory testing	7 (2.6)	12 (3.2)
	Same-day specialist referral	7 (2.6)	10 (2.9)
	Discharged	206 (76.3)	296 (80.0)
	No data on disposition	49 (18.1)	54 (14.5)

^a^ *N* = determined by complete triage data

Overall, admission rates to hospital were highest among those patients triaged as *emergency* cases either by the EP’s (21.4%) or the clinicians (83.3%). Fewer patients triaged as *priority* by either EPs or clinicians were admitted to hospital (4.6 and 14.9% respectively), however these patients had the highest prevalence of same day lab testing and/or specialty referral (see Table 2). Rates for discharge without admission, investigation or referral were highest amongst those patients triaged as *routine* either by expert patients and clinicians (76.3 and 80.0% respectively).

## Discussion

Task-shifting of services beyond professional health cadres to lay health workers has been an important strategy employed by governments and non-governmental organizations working in the context of the HIV epidemic in southern Africa to mitigate human resources for health bottlenecks [26–28]. Malawi, as a low-resource country dealing with a high prevalence of HIV, has a long history of formally incorporating lay-health care workers into their health system most notably through the establishment of a lay health worker cadre known as the health surveillance assistant [29]. In the context of the HRH constraints amplified by the rapid roll out of HIV services, informal lay-health worker strategies targeting HIV-related services have been employed by partners and endorsed by the MOH [24, 30–32]. Though there is some literature demonstrating that task-shifting can improve quality of care [33–38] worse outcomes can also occur when tasks are shifted to inadequately trained personnel [39, 40].

This study is the first in the literature to formally evaluate the effectiveness and safety of task-shifting of triage to the subset of lay health workers, known as “expert patients” in an ambulatory low-resource setting. The notion and role of the “expert” in this context departs from general conceptualizations of the same terminology whereby “expertise” is premised on the lived experience of a specific condition and on the ability to manage it successfully [3]. In contrast, in the Malawi HIV program setting, the identity of “expert patients” is perceived as an acquired professional identity and linked to acquired skills, information, knowledge and training that contributes to a quasi-professional identity linked to the task-shifting of clinical tasks that has happened as a mitigation strategy for HRH constraints with large scale antiretroviral therapy roll-out [41].

This intervention was based on the ETAT Course as developed by the WHO, which advocates for the involvement of lay health care workers to provide the initial assessment of patients for triage purposes in low-resource health care facilities [42]. To date, studies published on the effectiveness of ETAT have primarily involved formal health care workers, including doctors and nurses, in its implementation [43–45]. One study involving pediatric patients at a tertiary referral centre in Malawi, evaluated task-shifting of a recently developed inpatient monitoring and triage tool (ITAT) to a parallel lay health care worker cadre that were trained as “vital signs assistants” [46].

Our study provides reassurance that in the context of adequate training and ongoing supervision, task-shifting this clinical skill-set of triage to lay health care workers does not necessarily lead to less accurate triaging. While triaging scores did not always match exactly between EPs and clinicians, we report a trend among EPs to “over-triage” patients, meaning they often assigned a falsely elevated score to patients giving them a higher sensitivity and negative predictive value. We identified a priori to this study that the most important value of this system was that EPs would not miss critically ill patients needing immediate or expedited attention in the clinic and as such feel that this trend towards over-triage is in fact a protective trend for clinic patients and acceptable in practice. This is similar to findings from the ITAT intervention for a pediatric inpatient ward, which saw an increase in the number of clinician notifications with the capacitation of trained lay health workers to conduct triage and vital signs assessment [46].

We confirm this task is safely within the scope of practice possible for lay health care workers.

In a busy, ambulatory ART clinic that services a wide range of adult and pediatric patients at different stages of their HIV disease, an effective system to triage those arriving, as early as possible, with either life-threatening illness or illness requiring admission to the hospital is vital. We further confirm the ability of this task-shifted system to identify patients who required extra services (i.e. laboratory testing, same-day consultation and/or admission) when they arrive at the clinic, allowing for earlier identification to clinicians for disposition assessment, as the rates of these events was correlated to the triage score.

This study was designed as a program evaluation and we are unable to further comment on outcomes of patients. The literature in similar settings, however, supports a reduction in mortality based on the implementation of improved triage using ETAT principles in African settings [43–46]. We have shown that patients who were acutely ill were adequately identified by our lay health care workers and received expedited referral, investigations or admission early in the day.

In the context of an exacerbation of the HRH crisis being potentiated by the successful large-scale roll out of HIV services, this evaluation of triage quality adds to the growing body of literature supporting task-shifting of some clinical tasks, in a well-trained and sustainably supervised program, to lay health worker cadres [24, 30–32]. Larger studies will be needed to evaluate if task-shifting of triage can lead to improved patient outcomes such as mortality.

### Limitations

We recognize several limitations to this study. First, clinical officers were used as the gold standard for triage scores, however there may be differences in their range of expertise and our data does not allow us to assess the inter-rater reliability of our clinicians. Furthermore, the study is limited to immediate disposition outcomes and cannot comment further on overall impact on morbidity or mortality for those patients triaged to early assessment and intervention.

## Conclusion

In an era of ongoing human resources for health crises in many low-income countries, task-shifting to lay health care cadres is one potential strategy for improving quality of services for patients. We show that a lay health care cadre, namely “expert patients”, in an low-resource ambulatory ART clinic in Malawi can provide safe and effective triaging to improve the identification and early assessment of acutely ill patients. We encourage further development and evaluation of innovative programmes to support the incorporation of lay health care workers into similar settings.

## Additional file

Additional file 1: Work Aid for EP Triage Training. Description of data: A work aid summarizing the triage process and training for Expert Patients, modified from the WHO ETAT training. This work aid was also translated into Chichewa (the local language). (PPTX 99 kb)

## Abbreviations

ART: Antiretroviral therapy
EP: Expert patient
EPs: Expert patients
ETAT: Emergency Triage Assessment and Treatment
HIV: Human Immunodeficiency Virus
HRH: Human resources for health
ITAT: Inpatient monitoring and triage tool
LICs: Low income countries
MOH: Ministry of Health
NGO: Non-governmental organization
NPV: Negative predictive value
PLHIV: People living with human immunodeficiency virus
PPV: Positive predictive value
WHO: World Health Organization

## References

[1] Kober K and Van Damme W. Expert patients and AIDS care: a literature review on expert patient programmes in high-income countries, and an exploration of their relevance for HIV/AIDS care in low-income countries with severe human resource shortages. Available at: http://www.equinetafrica.org/sites/default/files/uploads/documents/KOBaids.pdf. Accessed 8 May 2017.

[2] Hermann K, van Damme W, Pariyo GW (2009). Community health workers for ART in sub-Saharan Africa: learning from experience – capitalizing on new opportunities. Hum Resour Health.

[3] Kielman K, Cataldo F (2010). Tracking the rise of the “expert patient” in evolving paradigms of HIV care. AIDS Care.

[4] Taylor D, Bury M (2007). Chronic illness, expert patients and care transition. Sociol Health Illn.

[5] Sanjana P, Torpey K, Schwarzwalder A, Simumba C, Kasonde P, Nyirenda L, Kapanda P, Kakungu-Simpungwe M, Kabaso M, Thompson C (2009). Task-shifting HIV counselling and testing services in Zambia: the role of lay counsellors. Hum Resour Health.

[6] Bedelu M, Ford N, Hilderbrand K, Reuter H (2007). Implementing antiretroviral therapy in rural communities: the Lusikisiki model of decentralized HIV/AIDS care. J Infect Dis.

[7] Chan AK, Mateyu G, Jahn A (2010). Outcome assessment of decentralization of antiretroviral therapy provision in a rural district of Malawi using an integrated primary care model. Trop Med Int Health.

[8] Callaghan M, Ford N, Schneider H (2010). A systematic review of task- shifting for HIV treatment and care in Africa. Hum Resour Health.

[9] Fulton BD, Scheffler RM, Sparkes SP, Auh EY, Vujicic M, Soucat A (2011). Health workforce skill mix and task shifting in low income countries: a review of recent evidence. Hum Resour Health.

[10] Zachariah R, Ford N, Philips M, Lynch S, Massaquoi M, Janssens V, Harries AD (2009). Task shifting in HIV/AIDS: opportunities, challenges and proposed actions for sub-Saharan Africa. Trans R Soc Trop Med Hyg.

[11] Joseph JK, Rigodon J, Cancedda C (2012). Lay health workers and HIV care in rural Lesotho: a report from the field. AIDS Patient Care STD.

[12] Crigler L, Wendo D, Guyer A, Nabwire J. Task shifting in HIV/AIDS service delivery: an exploratory study of expert patients in Uganda. Research and evaluation report. Published by the USAID Health Care Improvement Project. Bethesda: University Research Co., LLC (URC); 2011. Available at: https://www.usaidassist.org/sites/assist/files/uganda_expert_patients_nov11.pdf. Accessed 7 May 2017.

[13] Kyakuwa M, Hardon A, Goldstein Z (2012). “The Adopted Children of ART”: expert clients and role tensions in ART provision in Uganda. Med Anthropol.

[14] Dhlamini L, Knight L, van Rooyen H, van Heerden A, Jane Rotheram-Borus M (2012). Qualitative interviews with mentor mothers living with HIV: potential impacts of role and coping strategies. J Int AIDS Soc.

[15] Hussen SA, Tsegaye M, Argaw MG, Andes K, Gilliard D, del Rio C (2014). Spirituality, social capital and service: factors promoting resilience among Expert Patients living with HIV in Ethiopia. Glob Public Health.

[16] Chang LW, Kagaayi J, Nakigozi G (2010). Effect of peer health workers on AIDS care in Rakai, Uganda: a cluster-randomized trial. PLoS One.

[17] Arem H, Nakyanjo N, Kagaay K (2011). Peer health workers and AIDS care in Rakai, Uganda: a mixed methods operations research evaluation of a cluster-randomized trial. AIDS Patient Care STDS.

[18] Wools-Kaloustian KK, Sidle JE, Selke HM (2009). A model for extending antiretroviral care beyond the rural health centre. J Int AIDS Soc.

[19] Selke HM, Kimaiyo S, Sidle JE (2010). Task-shifting of antiretroviral delivery from health care workers to persons living with HIV/AIDS: clinical outcomes of a community-based program in Kenya. J Acquir Immune Defic Syndr.

[20] Pearson CR, Micek MA, Simoni JM (2007). Randomized control trial of peer-delivered, modified directly observed therapy for HAART in Mozambique. J Acquir Immune Defic Syndr.

[21] National Statistics Office. 2008 Population and Housing Census. Lilongwe: Government of Malawi; 2008. Available at: http://www.nsomalawi.mw/images/stories/data_on_line/demography/census_2008/Main%20Report/Census%20Main%20Report.pdf. Accessed 7 May 2017.

[22] Ministry of Health (2008). HIV and syphilis sero-survey and National HIV prevalence and AIDS estimates report 2007.

[23] Dignitas International Monitoring and Evaluation Department (2010). Quarterly monitoring and evaluation report: 2010 Q2.

[24] Tenthani L, Cataldo F, Chan AK (2012). Involving expert patients in antiretroviral treatment provision in a tertiary referral hospital HIV clinic in Malawi. BMC Health Serv Res.

[25] World Health Organization. Emergency Triage Assessment and Treatment (ETAT) course. Available at: http://apps.who.int/iris/bitstream/10665/43386/1/9241546875_eng.pdf. Accessed 7 May 2017.

[26] Wouters E, van Damme W, van Rensburg D, Masquillier C, Meulemans H (2012). Impact of community-based support services on antiretroviral treatment programme delivery and outcomes in resource-limited countries: a synthetic review. BMC Health Serv Res.

[27] Decroo T, Rasschaert F, Telfer B, Remartinez D, Laga M, Ford N (2013). Community-based antiretroviral therapy programs can overcome barriers to retention of patients and decongest health services in sub-Saharan Africa: a systematic review. Int Health.

[28] Kredo T, Adeniyi FB, Bateganya M, Pienaar ED (2014). Task shifting from doctors to non-doctors for initiation and maintenance of antiretroviral therapy. Cochrane Database Syst Rev.

[29] Smith S, Deveridge A, Berman J (2014). Task-shifting and prioritization: a situational analysis examining the role and experiences of community health workers in Malawi. Hum Resour Health.

[30] Bemelmans M, Van den Akker T, Ford N, Philips M, Zachariah R, Harries A, Schouten E, Mwagomba B, Massaquoi M (2010). Providing universal access to antiretroviral therapy in Thyolo, Malawi through task shifting and decentralization of HIV/AIDS care. Trop Med Int Health.

[31] McCollum ED, Preidis GA, Kabue MM (2010). Task shifting routine inpatient pediatric HIV testing improves program outcomes in urban Malawi: a retrospective observational study. PLoS One.

[32] LaCourse SM, Chester FM, Matoga M (2015). Implementation and operational research: implementation of routine counselor-initiated Opt-Out HIV testing on the adult medical ward at Kamuzu Central Hospital, Lilongwe, Malawi. J Acquir Immune Defic Syndr.

[33] Dovlo D (2004). Using mid-level cadres as substitutes for internationally mobile health professionals in Africa. A desk review. Hum Resour Health.

[34] Huicho L, Scherpbier RW, Nkowane AM, Victora CG (2008). How much does quality of child care vary between health workers with differing durations of training? An observational multicountry study. Lancet.

[35] Chilopora G, Pereira C, Kamwendo F (2007). Postoperative outcome of caesarean sections and other major emergency obstetric surgery by clinical officers and medical officers in Malawi. Hum Resour Health.

[36] Haines A, Sanders D, Lehmann U (2007). Achieving child survival goals: potential contribution of community health workers. Lancet.

[37] Lewin SA, Dick J, Pond P (2005). Lay health workers in primary and community health care. Cochrane Database Syst Rev..

[38] Lehmann U, Van Damme W, Barten F, Sanders D (2009). Task shifting: the answer to the human resources crisis in Africa?. Hum Resour Health.

[39] Shutt LMM (1994). Midterm review of the Tanzania Family Planning Services Support (FPSS) project (621–0173). POPTECH Report No. 94-011- 015.

[40] Ofori-Adjei D, Arhinful DK (1996). Effect of training on the clinical management of malaria by medical assistants in Ghana. Soc Sci Med.

[41] Cataldo F (2013). Patient-centered innovations in HIV care and transfer of “knowledge”: the case of expert patients at Dignitas International, Zomba.

[42] Gove S, Tamburlini G, Molyneux E, Whitesell P, Campbell H (1999). Development and technical basis of simplified guidelines for emergency triage assessment and treatment in developing countries. WHO Integrated Management of Childhood Illness (IMCI) Referral Care Project. Arch Dis Child.

[43] Robison JA, Durand C, Nosek CA (2012). Decreased pediatric hospital mortality after an intervention to improve emergency care in Lilongwe, Malawi. Pediatrics.

[44] Clark M, Spry E, Daoh K, Baion D, Skordis-Worrall J (2012). Reductions in inpatient mortality following interventions to improve emergency hospital care in Freetown, Sierra Leone. PLoS One.

[45] Molyneux E, Ahmad S, Robertson A (2006). Improved triage and emergency care for children reduces inpatient mortality in a resource-constrained setting. Bull World Health Organ.

[46] Olson D, Preidis GA, Milazi R (2013). Task-shifting an inpatient triage, assessment and treatment program improves the quality of care for hospitalized Malawian children. Trop Med Int Health.

