# Supplementary material for: Task shifting of triage to peer expert informal care providers at a tertiary referral HIV clinic in Malawi: a cross-sectional operational evaluation
Source: BMC Health Serv Res. 2017 May 9;17:341. doi: 10.1186/s12913-017-2291-3 (PMC5423418; doi:10.1186/s12913-017-2291-3)
Supplement: Additional file 1: — Work Aid for EP Triage Training. Description of data: A work aid summarizing the triage process and training for Expert Patients, modified from the WHO ETAT training. This work aid was also translated into Chichewa (the local language). (PPTX 99 kb) [file 12913_2017_2291_MOESM1_ESM.pptx]

## Slide 1
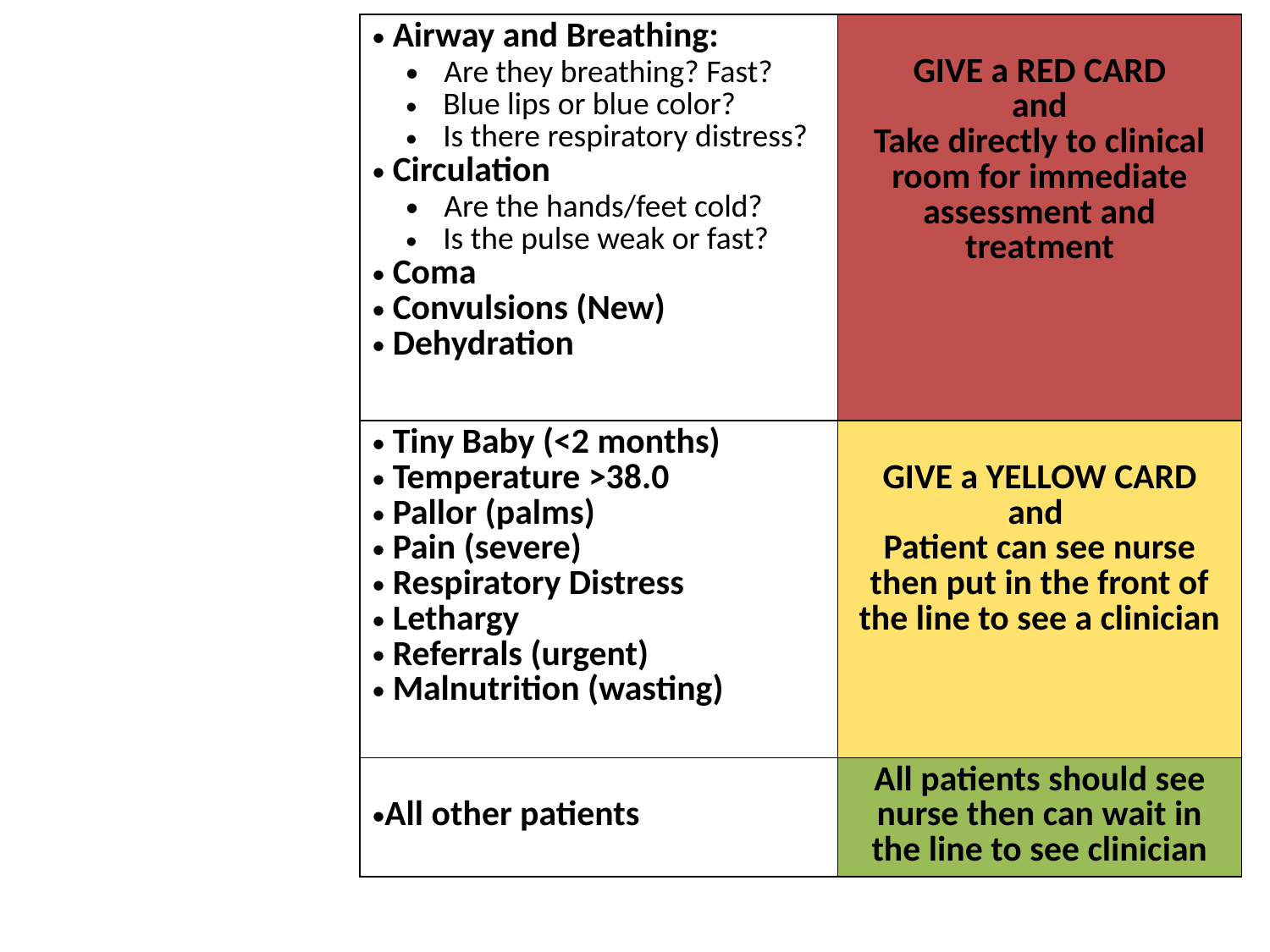

| Airway and Breathing: Are they breathing? Fast? Blue lips or blue color? Is there respiratory distress? Circulation Are the hands/feet cold? Is the pulse weak or fast? Coma Convulsions (New) Dehydration | GIVE a RED CARD and Take directly to clinical room for immediate assessment and treatment |
| --- | --- |
| Tiny Baby (<2 months) Temperature >38.0 Pallor (palms) Pain (severe) Respiratory Distress Lethargy Referrals (urgent) Malnutrition (wasting) | GIVE a YELLOW CARD and Patient can see nurse then put in the front of the line to see a clinician |
| All other patients | All patients should see nurse then can wait in the line to see clinician |
